# Temporal redistribution of inhibition over neuronal subcellular domains underlies state-dependent rhythmic change of excitability in the hippocampus

**DOI:** 10.1098/rstb.2012.0518

**Published:** 2014-02-05

**Authors:** Peter Somogyi, Linda Katona, Thomas Klausberger, Bálint Lasztóczi, Tim J. Viney

**Affiliations:** 1 Medical Research Council, Anatomical Neuropharmacology Unit, Department of Pharmacology, Oxford University, Mansfield Road, Oxford OX1 3TH, UK; 2 Center for Brain Research, Department of Cognitive Neurobiology, Medical University of Vienna, Vienna 1090, Austria

**Keywords:** oscillation, interneuron, inhibition, synapse, GABA

## Abstract

The behaviour-contingent rhythmic synchronization of neuronal activity is reported by local field potential oscillations in the theta, gamma and sharp wave-related ripple (SWR) frequency ranges. In the hippocampus, pyramidal cell assemblies representing temporal sequences are coordinated by GABAergic interneurons selectively innervating specific postsynaptic domains, and discharging phase locked to network oscillations. We compare the cellular network dynamics in the CA1 and CA3 areas recorded with or without anaesthesia. All parts of pyramidal cells, except the axon initial segment, receive GABA from multiple interneuron types, each with distinct firing dynamics. The axon initial segment is exclusively innervated by axo-axonic cells, preferentially firing after the peak of the pyramidal layer theta cycle, when pyramidal cells are least active. Axo-axonic cells are inhibited during SWRs, when many pyramidal cells fire synchronously. This dual inverse correlation demonstrates the key inhibitory role of axo-axonic cells. Parvalbumin-expressing basket cells fire phase locked to field gamma activity in both CA1 and CA3, and also strongly increase firing during SWRs, together with dendrite-innervating bistratified cells, phasing pyramidal cell discharge. Subcellular domain-specific GABAergic innervation probably developed for the coordination of multiple glutamatergic inputs on different parts of pyramidal cells through the temporally distinct activity of GABAergic interneurons, which differentially change their firing during different network states.

## 1. Introduction

Exploration of neuronal activity in the hippocampus [1] and lesion studies [2] led to the conclusion that the hippocampus is involved in the encoding and recall of spatial information [3–5], one form of processing temporal sequences of events. The dorsal hippocampus is intricately connected to subcortical areas and a temporal cortical system, including the perirhinal, the entorhinal, the retrosplenial and the subicular cortices, which receive polysynaptic sensory-motor information and produce navigation-related activity. The most salient cellular representations include the place cells of the hippocampus [5] and head-direction cells [6], grid cells [7], border [8] and boundary vector cells [9] in other areas. A common feature of these spatial tuning specificities is that while some of these cells discharge, others in the same cortical area, even adjacent to active cells, are silent. Inhibitory interactions between cell assemblies via local GABAergic (producing gamma-aminobutyric acid) interneurons probably make a strong contribution to the emergence of these specificities [10–12].

During spatial navigation, or the offline replay of spatial representations, neuronal activity in the temporal lobe is rhythmic, showing synchronization at various frequencies. Rhythmic changes in the extracellular electrical potential show that ion currents are spatially organized and synchronous through cell membranes; the greatest contributors being glutamatergic and GABAergic synaptic activity [13]. Rhythmic synchronization occurs in the theta (4–12 Hz) gamma (30–150 Hz) and sharp wave-related ripple (SWR; 120–200 Hz) bands, which are related to well-defined behaviours. The firing of information-coding principal cells, such as stellate cells of the entorhinal cortex, pyramidal cells in all areas of the system and granule cells of the dentate gyrus, is phase-related to these oscillations, reflecting their fluctuating excitability. One major contributor to neuronal excitability is intracortical inhibition produced by local GABAergic interneurons that are also phase locked to oscillations in complex ways [14–19]. Here, we explore how hippocampal GABAergic neuronal activity may change the excitability of pyramidal cells during theta and high-frequency ripple oscillations, two rhythms thought to represent key stages of navigation-related neuronal activity. We use the action potential output of identified types of interneuron with known sites of axonal termination as a surrogate of predicting GABA receptor activation on the postsynaptic cells, although we are aware that the short- and long-term plasticity of GABAergic synapses and the firing pattern influences the effect of presynaptic spikes [20–22]. In the hippocampus, some GABAergic neurons fire phase locked also to gamma oscillations [23], and are a key component of the mechanism generating gamma activity, but owing to space restriction their role in gamma oscillations is not discussed in detail here.

The diversity of interneurons, or non-principal cells, was recognized from their distinct shapes well before the identification of GABA and the other signalling molecules that they selectively express [24,25]. Specific types of interneuron recognized by their shapes from Golgi impregnation [26] or intracellular injection of tracer molecules [27] reflect distinct synaptic relationships to pyramidal cells and can be further specified by the selective localization of molecules involved in intercellular signalling such as neuropeptides, receptors and calcium-binding proteins [28–30]. Crucially, for behaviour-related neuronal dynamics, interneuron types defined by their distinct synaptic relationships show remarkable firing specificity also during different network states *in vivo* [17,31]. In the CA1 area, more than 20 types of interneuron have been defined, 15 of which have also been recorded *in vivo*, and some of the same cell types have also been recorded and identified in the upstream CA3 area [32,33], providing an opportunity to compare roles. The differentiation of neuronal types has required labelling of the cells, which until recently was possible only during urethane anaesthesia, which retains many of the network activity patterns, such as theta and ripple oscillations, albeit at a reduced frequency. More recently, it has become possible to record and label interneurons in non-anaesthetized rodents [34,35], and we provide some comparisons between anaesthetized and non-anaesthetized activities for the same cell types.

Many cortical neuron network models prominently feature ‘perisomatic’ inhibition, a collective term of convenience for the action of synaptic terminals and GABA_A_ receptor activation on the soma, the proximal dendrites and the axon initial segment. However, in the light of the evidence that adjacent GABAergic synapses can have completely opposite temporal dynamics *in vivo*, depending on the identity of the GABA-releasing neuron [30,31], the term no longer makes sense without specifying the GABA-releasing cell type. Similarly, dendrites are innervated by GABA-releasing neurons with highly differentiated and specific temporal dynamics [36]. Accordingly, lumping dendritic innervation by interneurons into a single entity as ‘dendritic inhibition’ obscures the rules that may explain operational principles.

**Figure 1.**
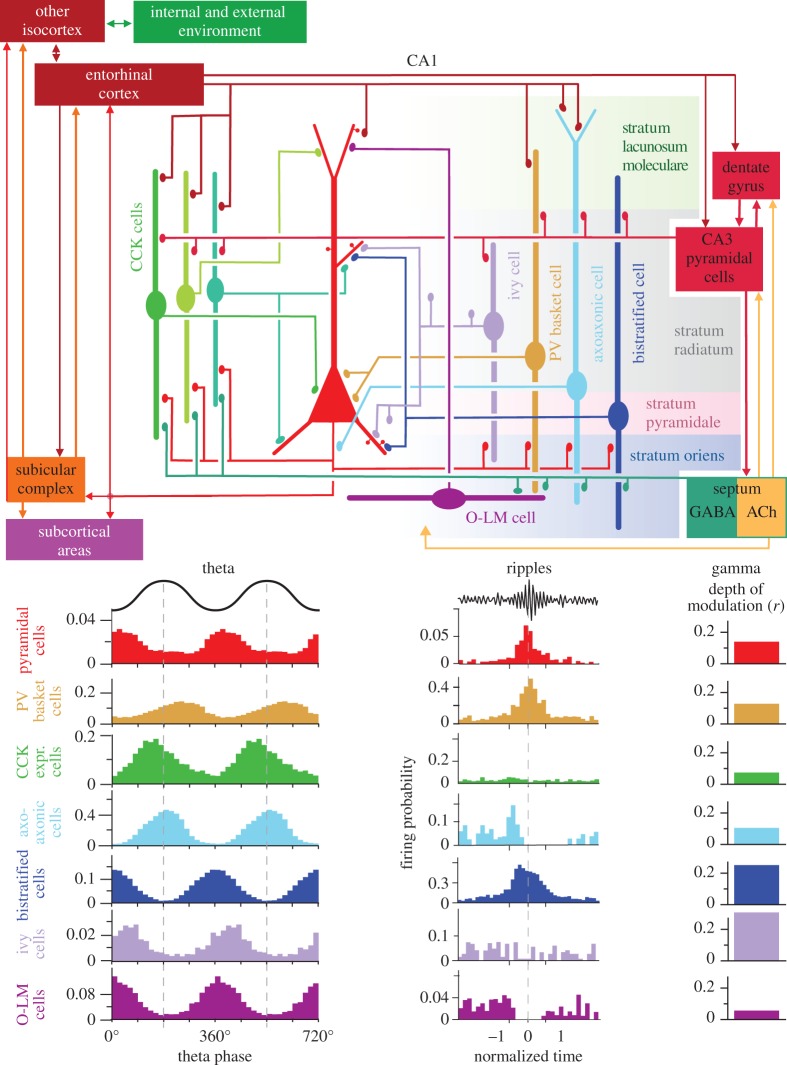
Schematic of the spatial and temporal relationships between pyramidal cells and eight types of GABAergic interneuron in the CA1 area. Top: the main synaptic connections of pyramidal cells (red, middle), three types of CCK-expressing cells (basket cell, perforant path-associated cell, Schaffer collateral-associated cell), ivy cells and PV-expressing basket, axo-axonic, bistratified and O-LM interneurons. The other 13 defined types of interneuron are not shown; ACh, acetycholine. The firing probability histograms are averages from several cells of the same type recorded in anaesthetized rats; note different scales for the *y*-axis and schematic LFP oscillations (black). During theta and ripple oscillations, interneurons innervating different domains of pyramidal cells fire with distinct patterns. Their firing is coupled to field gamma oscillations to varying degrees (averages of several cells of each type). The soma and dendrites are innervated in parallel by both CCK- or PV-expressing cells, which show different temporal dynamics. One key feature is that during theta, pyramidal cell firing probability is lowest at the peak of the pyramidal layer LFP, when axo-axonic cells innervating the axon initial segment fire maximally and the sum of CCK- and PV-expressing basket cell firing probability is also maximal. The cooperative action of these three GABAergic cell types suppresses pyramidal cell firing at the peak of theta. The dendrite-innervating cells show similar theta phase coupling, approximately counter-phased with the combined impact of perisomatic innervating cells. Bistratified and ivy cells innervating basal and small oblique pyramidal cell dendrites show the highest gamma coupling of their firing and most of their dendrites are in the input zone from CA3 pyramidal cells which fire strongly phase coupled to gamma oscillations in CA3. During ripple oscillations, GABA release to the axon initial segments from axo-axonic cells is withdrawn, allowing maximal pyramidal cell discharge synchronized by PV-expressing basket and bistratified cells. Connections among interneurons are not shown for clarity. (Data from [15,37–40].)

Distinct types of interneuron specialize in innervating functionally different domains of pyramidal cells (figure 1). Only axo-axonic cells innervate the axon initial segment; the soma and proximal dendrites are innervated by at least three types of basket cell. The dendritic domain is innervated by at least 14 types of dendrite targeting cells, some of which localize their output synapses either in strata oriens and radiatum, such as bistratified cells, or on the most distal dendrites in stratum lacunosum moleculare such as oriens-lacunosum moleculare (O-LM) and neurogliaform cells [30]. Interestingly, all domains of pyramidal cells are innervated by a group of interneuron types expressing the calcium-binding protein parvalbumin (PV). In addition, all domains, except the axon initial segment, are also innervated by a distinct set of cholecystokinin-expressing (CCK) GABAergic cells. The PV- and CCK-expressing families of interneurons differ in their biophysical parameters, synaptic connectivity, neurochemical components and *in vivo* firing patterns [31,38,41]. A set of six types of GABAergic cell, in addition to innervating pyramidal cells and interneurons in the hippocampus, also project to extrahippocampal areas such as the septum, subiculum, indusium griseum, retrosplenial cortex and the entorhinal cortex [37,42,43]. Interneurons are interconnected in complex ways [44], and there is a set of specialized interneurons innervating other interneuron types mostly or exclusively [29]. It is not yet clear whether all types of CA1 pyramidal cell receive input from all of the interneurons innervating pyramidal cells.

## 2. Theta oscillation and interneuron firing

Several models of the spatial navigation system suggest a role for rhythmic change in principal cell activity at theta frequencies [45–49]. Indeed, the frequency and amplitude of local field potential (LFP) theta oscillations are strongly modulated by the speed of movement, with speed positively correlated to theta frequency [50,51]. During theta oscillations, GABA-mediated inhibition rhythmically changes in pyramidal cells [52–56]. The overall population of active pyramidal cells fires the most action potentials per theta cycle just after the trough of the LFP theta cycle recorded in the pyramidal layer, indicating the highest excitability [57,58]. When the animal enters the place field of a given pyramidal place cell, the cell starts firing close to the peak of the extracellular theta field potential [58], when the overall population of pyramidal cells are strongly inhibited. As the animal traverses the place field, place cells fire with a systematic backward phase shift (phase precession) in their coupling to the theta LFP on consecutive theta cycles. Intracellular recordings show that place cells discharge when the theta-rhythmically changing membrane potential is most depolarized [59,60] and within the place field the intracellular oscillation is faster than the extracellular LFP theta oscillation [60]. The firing of simultaneously recorded place cells and interneurons can be either positively or negatively correlated [10,61,62] indicating diverse relationships and possibly different interneuron cell types. Positively correlated pairs of pyramidal cells and interneurons share place fields [10,61,62], whereas negatively correlated pairs may have complementary ones [10], and the spatial information content of interneuron firing is similar [63] or higher [14] than that of pyramidal cells. How GABAergic neurons, firing at different phases of the theta cycle and terminating on different domains of pyramidal cells, contribute to the intracellular membrane potential oscillation and the LFP theta is poorly understood.

**Figure 2.**
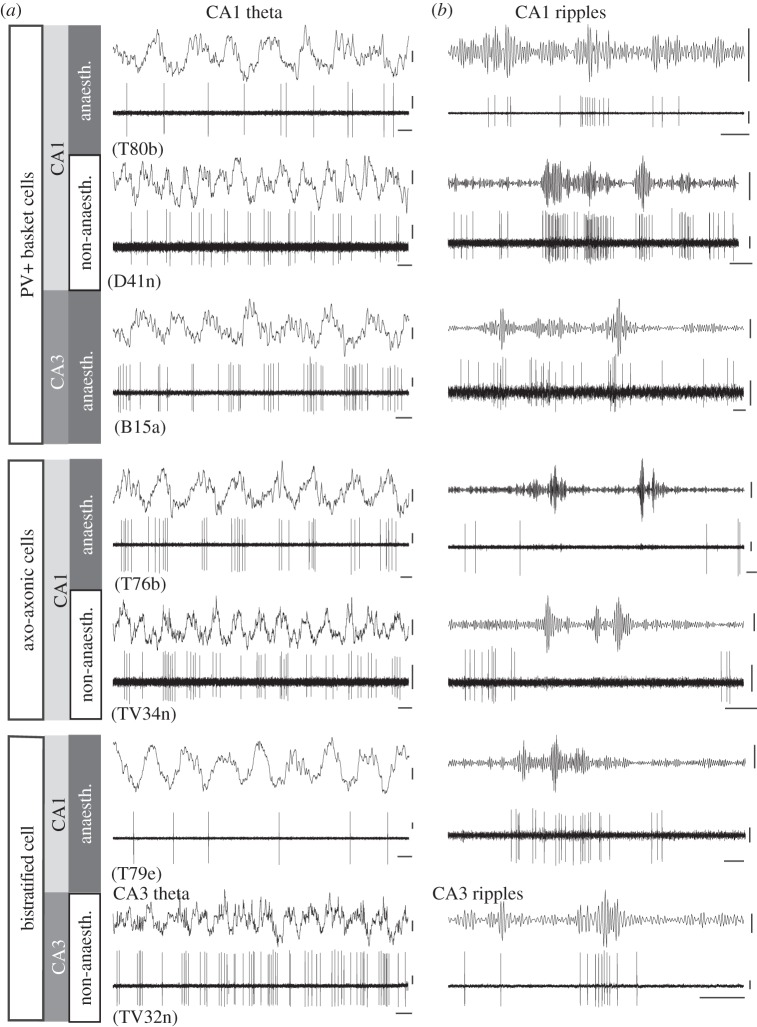
Network activity-related firing patterns of the three major parvalbumin-expressing GABAergic neurons of stratum pyramidale in areas CA1 and CA3 of rats, with or without anaesthesia. (*a*) Theta was recorded in the pyramidal layer of CA1, except for the CA3 bistratified cell (TV32n, bottom). In CA1, PV-positive basket cells fire on the descending phase of CA1 pyramidal layer theta. Axo-axonic cells fire just after the theta peak, earlier than basket cells, and bistratified cells later, around the trough, at the highest firing probability of pyramidal cells. Note the increase in the frequency of theta oscillations without anaesthesia, but the nearly constant phase relationships in spite of increase in firing rates. (*b*) During SWRs, basket and bistratified cells strongly increase their firing rate, phase locked to the local oscillation, but axo-axonic cells reduce firing either with or without anaesthesia. Theta field potential, wide band; SWRs, LFP band-pass filtered (90–140; non-anaesthetised, 90–200 or 130–230 Hz) for ripple activity. Scale bars: 0.1 s; action potentials and field potential theta, 0.2 mV; ripples, 0.1 mV. (Data from [15,33,34,40], CA3 bistratified cell without anaesthesia (TJ Viney 2013, unpublished data).)

**Figure 3.**
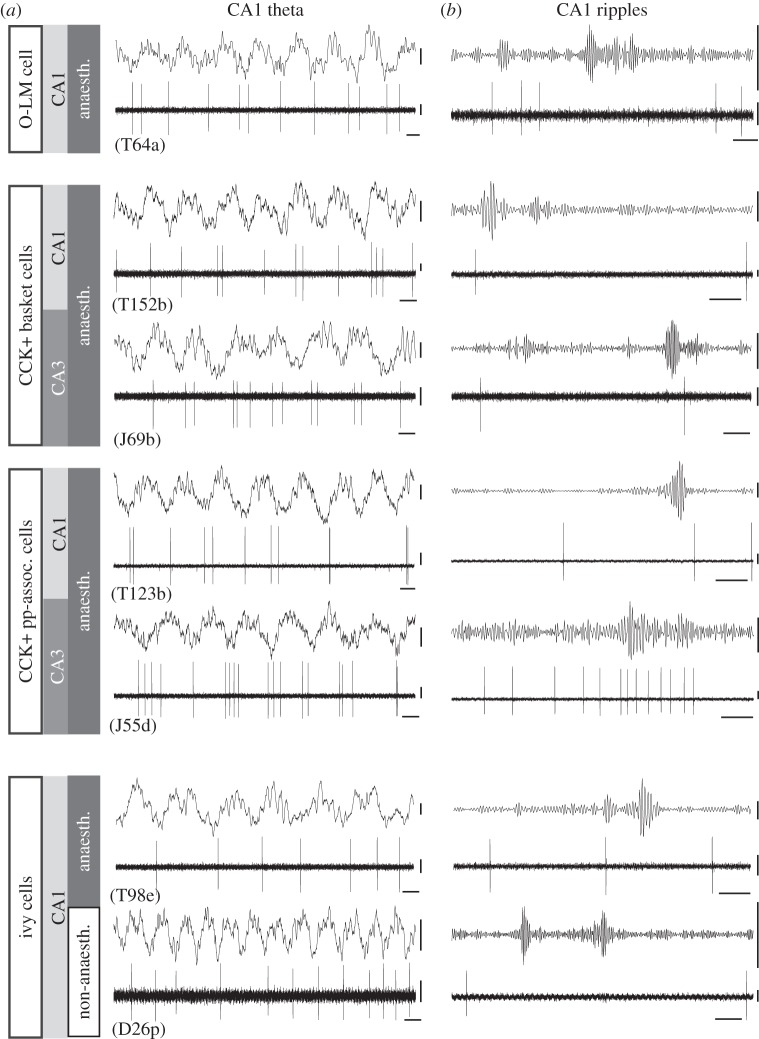
Firing patterns related to network activity of CCK-expressing GABAergic neuronal types that collectively innervate the entire somato-dendritic domain of pyramidal cells and of dendrite-innervating O-LM and ivy cells in areas CA1 and CA3 of rat hippocampus. (*a*) Theta was recorded in the pyramidal layer of CA1. Ivy cells have been reported with and without anaesthesia; note the increase in theta frequency without anaesthesia, and the constant theta firing phase. All cell types, except basket cells in CA1 discharge with maximal probability near the trough of CA1 pyramidal layer theta, when pyramidal cells are most active in both areas under anaesthesia. (*b*) During SWR oscillations detected in CA1, on average, most cell types shown here in both areas do not change their firing rate; the exception is the perforant path-associated (pp-assoc.) cells in CA3, which increase their discharge. LFP, as for figure 2. Scale bars: 0.1 s; action potentials and field potential theta, 0.2 mV; ripples, 0.1 mV. (Data from [15,32,34,38,65].)

Hippocampal GABAergic interneurons, which provide most of the inhibitory input to pyramidal cells, fire on average at higher rates than pyramidal cells and are also phase locked to the theta rhythm both in freely moving animals [64] and in urethane-anaesthetized preparations (figures 2 and 3). All interneuron types recorded under anaesthesia showed significant theta-modulated firing [31], which we will use below as a surrogate to predict GABA released to the domain of pyramidal cells innervated by a given interneuron type. Most of the time, the rhythmically firing interneuron has a stable phase relationship to the theta field potential, but in awake animals during movement some interneurons also show phase precession for short periods [14,45,61].

In CA1 during anaesthesia, PV-expressing basket cells fire with highest probability on the descending (figures 1 and 2), whereas CCK-expressing basket cells fire on the ascending (figures 1 and 3) phase of theta oscillations in the pyramidal cell layer. The weighted sum of their action potential output is highest at the peak of extracellular theta in the pyramidal layer and lowest at the trough of the LFP [38], thus correlating with the lowest and highest firing probability of pyramidal cells, respectively. However, the firing of bistratified and O-LM cells is maximal at the trough of theta (figures 1–3), as it is for other interneurons that innervate dendrites, such as double projection (to both septum and retrohippocampal areas), hippocampo–retrohippocampal and ivy cells. Accordingly, as GABA release to the soma and proximal dendrites decreases around the trough of theta, GABA release to the more distal dendrites increases. Perhaps the strongest influence on the rhythmic change in pyramidal cell firing probability during theta is provided by the axo-axonic cell, which exclusively targets the axon initial segment, the site of action potential generation. The firing of axo-axonic cells is strongly phase coupled close to the pyramidal layer theta LFP peak in both anaesthetized and non-anesthetized rats (figures 1 and 2), when pyramidal cells fire least, suggesting their key inhibitory role [15]. Interestingly, another GABAergic neuron that is phase locked to theta pyramidal layer LFP peak is the neurogliaform cell innervating the most distal dendrites and showing slow dynamics [66]; thus, the two poles of pyramidal cells receive GABA at the same time, but with very different effects [67]. Both the axo-axonic cells and the neurogliaform cells have extensive dendritic arbours in stratum lacunosum moleculare. This stratum is innervated by the entorhinal cortex, which may contribute to the theta phase preference of these cells, because, in this layer, at the peak of pyramidal layer theta LFP, there is a current sink suggesting glutamate receptor activation [4]. In summary, the tracing of the GABA-releasing terminals of distinct interneurons with different preferred theta firing phases reveals *a rhythmic and sequential redistribution of GABA receptor activation over different subcellular domains of pyramidal cells during each theta cycle*.

How well can we extrapolate from oscillatory phase preferences under urethane anaesthesia to the network role of interneurons in the freely navigating animal? Under anaesthesia, the firing rates are lower, and theta frequency is around 4–5 Hz (figures 2 and 3), the low end of the range observed in drug-free animals, but the preferred firing phase may not change much. Consistent with this assumption, using the distinct theta firing phase of identified PV-expressing basket, axo-axonic, bistratified, O-LM and CCK-expressing interneurons reported earlier, Czurkó *et al*. [16] isolated four groups of interneuron recorded with tetrodes in foraging rats. Furthermore, using novel labelling techniques, three types of identified GABAergic interneuron have been reported in drug-free rodents [34,35]. In freely moving rats, PV-expressing basket cells fired at the descending phase of dorsal CA1 pyramidal layer theta as in anaesthetized rats in spite of the doubling of theta frequency and a higher firing rate than under anaesthesia (figure 2). Similarly, in head-fixed mice running on a suspended ball, PV-expressing basket cells in CA1 also fired at the descending phase [35]. In drug-free animals, the dendrite-innervating ivy cells [34] and O-LM cells [35] fired at theta trough, as in anaesthetized rats. Although more work is clearly needed, it appears that urethane anaesthesia, which has been necessary for stability in order to achieve labelling of the recorded cells, does not change firing phase of at least some identified interneurons during theta oscillations. An axo-axonic cell in CA1 recorded in a freely moving rat (figure 2) fired similarly to putative axo-axonic cells recorded by tetrodes [16], and to unidentified interneurons in freely moving mice termed theta driving cells [68], but with a mean theta phase slightly forward shifted relative to axo-axonic cells recorded under anaesthesia [15].

Theta oscillations modulate the amplitude of gamma oscillations in the hippocampus [23,69], which are involved in cognitive processes. The role of interneurons in general [70], and that of PV-expressing basket cells in particular [71,72], is well established in relation to gamma oscillations evoked *in vitro*. However, the contributions of different cell types have been difficult to define *in vivo* [69,73,74]. The tuning of hippocampal interneurons to gamma oscillations *in vivo* is cell-type specific (figure 1) [39] with particularly strong coupling observed for bistratified [39] and CA3 PV-expressing basket cells [33], with different types of CCK/CB1R expressing interneurons having differential gamma frequency dependence [32]. Some types of the latter are strongly coupled to both slow and fast gamma oscillations, but basket cell and interneurons innervating proximal dendrites are weakly coupled only to fast (50–100 Hz) gamma [32].

In summary, the cooperative increased firing of axo-axonic cells, together with significant firing of PV- or CCK-expressing basket cells at the pyramidal layer theta peak, decreases firing probability of pyramidal cells. To the contrary, the cessation of axo-axonic cell firing and a decrease in basket cell firing lowers inhibition on the axon initial segment, the soma and proximal dendrites around the trough of theta creating a window of higher probability for action potential generation by pyramidal cells.

What produces theta rhythmic activity of interneurons at preferred phases? In addition to the theta rhythm of excitatory glutamatergic inputs, a major contributor is the rhythmic medial septal GABAergic input [75], which selectively innervates interneurons [76]. Septal rhythmic cells show several preferred theta phases [77], and it was suggested that each might innervate only certain types of hippocampal interneuron [78]. However, although many medial septal neurons project to the hippocampus, the axon of no recorded septal neuron has been shown to innervate the hippocampus, and the medial septum also innervates other cortical and subcortical areas.

## 3. Sharp wave-associated ripple oscillations and interneuronal firing

During slow wave sleep, long periods of awake immobility and consummatory behaviour, the hippocampus displays large amplitude irregular activity containing synchronous population burst of subsets of pyramidal cells and interneurons resulting in a fast oscillation in the pyramidal layer (120–200 Hz), the ripple [5]. As O'Keefe & Nadel [5, p. 151] described it, ‘the large negative slow wave is intimately associated with two other neuronal events: a sinusoidal ripple consisting of 4–10 waves with periods of 4–8 ms (see their Fig. 14), and a burst of firing in the theta units located in stratum pyramidale and oriens’. Their ‘theta units’ [64] were subsequently identified as GABAergic interneurons (see below). The SWRs facilitate cortico–hippocampal interaction, whereas subcortical structures are silent [79]. The CA1 population burst is driven by the synchronous discharge of CA3 pyramidal cells [80,81], resulting in a large negative extracellular potential indicative of a current sink in stratum radiatum, the sharp wave [18] (the depolarization caused by Schaffer collateral/commissural axon synapses). During SWRs lasting 30–120 ms, place cells discharge sequentially [82–84] either in the reverse [85] or forward direction [86,87] of their temporal order during awake navigation, depending on the behavioural context. Such ordered offline neuronal activity has been proposed as contributing to memory consolidation [88], predicts correct or incorrect subsequent behavioural choices [89], and its selective disruption impairs subsequent performance [90–92]. Furthermore, similar SWRs, during awake navigation [93,94], may contribute to forming memories of the route [87]. Although such a population burst represents the largest known synchronous hippocampal pyramidal cell activity, involving an estimated 10–20% of all pyramidal cells, total GABAergic input to pyramidal cells is actually increased during SWRs relative to the peri-ripple periods. Does this contradict Buzsaki's [80] hypothesis that the CA3 pyramidal cell population burst is a result of disinhibition of pyramidal cells? In view of the highly compartmentalized GABAergic innervation, the answer may lie in the location of a simultaneous but differential increase and decrease of GABA receptor activation on distinct parts of pyramidal cells. Indeed, some unidentified interneurons were inhibited during SWRs, whereas others were strongly activated during SWRs in sleep [19]. Thus, it is possible that GABAergic interneuronal activity contributes to both the selection of active pyramidal cells via disinhibition on some of their subcellular domains, while on other domains of the same cells rhythmic GABA receptor activation produces synchronization and temporal ordering of the cells by setting up windows of decreased firing probability during ripples. Do the activated or inhibited interneurons during SWRs belong to different cell types? Which domain(s) of the pyramidal cell is disinhibited and on which domains does the ripple frequency inhibition operates? We examine these questions below.

Klausberger *et al*. [15,38] recorded and identified interneurons during SWRs *in vivo* in CA1 (figures 1–3) under anaesthesia. They showed that PV-expressing basket and bistratified cells fired phase locked to SWRs, whereas PV-expressing axo-axonic and O-LM cells were inhibited. Basket cells fired phase-coupled to local ripples also in CA3 [33], but not to simultaneous ripples in CA1, which shows that these events are re-structured locally in each area of the temporal lobe [95].

Subsequent recording of PV-expressing basket cells without anaesthesia in CA1 of rats [34] and mice [35] confirmed that these cells increase their firing during SWRs and thus contribute to the generation of ripple-related pyramidal cell phasing. In CA1 of non-anaesthetized mice, O-LM cells fired during some SWRs [35], in contrast to data from anaesthetized rats [15] (figure 3). However, SWRs were recorded in awake, immobile mice, whereas the SWRs in anaesthetized rats probably are more similar to SWRs generated during slow wave sleep. Thus, it remains to be established whether O-LM cells are activated during SWRs in natural slow wave sleep. In addition to PV-expressing basket and bistratified cells, other GABAergic neurons that project to extrahippocampal areas such as trilaminar cells [96], and double projection cells [42], including hippocampo-septal cells, also strongly increase their firing during SWRs, and some are coupled to the ripple phase [42]. These neurons innervate the basal and proximal apical dendritic region of pyramidal cells in CA1, and thus act in cooperation with bistratified cells, and in addition provide inhibitory postsynaptic potentials (IPSPs) at ripple frequency to their distant target areas in the septum, subiculum and other retrohippocampal areas.

Other interneuron types do not appear to change their activity in spite of having dendritic arbours in the termination zone of CA3 pyramidal cells and also receiving local recurrent input from CA1 pyramidal cells. One of the cell types is the ivy cell (figures 1 and 3), whose activity was recorded in both anaesthetized and non-anaesthetized rats [34,65]. Some CCK-expressing cells also do not change their firing rate during SWRs (figures 1 and 3) in both CA1 [38] and CA3 [32], whereas others fire strongly during some SWRs but remain silent during others [32,38]. Basket cells expressing CCK are of particular interest, because they innervate the same subcellular domain as do the strongly firing PV-expressing basket cells, yet they show very different temporal dynamics. During some SWRs, both types of basket cell may release GABA to the pyramidal somata in CA1, but overall PV-expressing basket cells dominate. The majority of CCK-expressing GABAergic cells in both CA1 [38] and CA3 [32] in general fire at a low rate during non-theta states under anaesthesia (figure 3), so their role during SWRs remains to be established during drug-free behaviour. Nerve terminals of CCK-expressing GABAergic neurons are richly endowed with CB1 cannabinoid receptors [20], which mediate retrograde suppression of GABA release by strongly depolarized postsynaptic neurons, such as firing pyramidal cells. As a result, the strongly firing neuron is likely to be disinhibited from the influence of CCK-expressing interneurons [41,97], which innervate the entire somato-dendritic domain. Is there a specific subcellular part of the pyramidal cell that is specifically disinhibited during SWRs?

Axo-axonic cells innervate the axon initial segment of pyramidal cells, activate GABA_A_ receptors [27] and were suggested to have an inhibitory role [98]. In brain slices *in vitro*, neocortical and hippocampal axo-axonic cells can also evoke pyramidal cell axonal firing [99–101], but this is likely to originate from axons whose local pyramidal cell has been sliced off or damaged. As described above, the firing of axo-axonic cells is negatively correlated with the firing probability of pyramidal cells *in vivo*. This was demonstrated also during SWRs, as axo-axonic cell firing was strongly inhibited in both anaesthetized [15] and non-anaesthetized rats (figure 2; TJ Viney 2013, unpublished data). This is surprising, because axo-axonic cells receive monosynaptic input from the CA1 pyramidal cells [102] and their main dendritic tree is in strata radiatum and oriens where the axons of the SWR initiating CA3 pyramidal cells terminate, and probably innervate axo-axonic cells directly. Both the somatic and the dendritic distribution of PV-expressing basket and axo-axonic cells are similar, thus the contrasting behaviour during SWRs is most likely due to a specific inhibitory input to axo-axonic cells which is highly active during SWRs, such as that from the PV-expressing basket and bistratified cells [31]. However, it is not yet known whether these cell types innervate axo-axonic cells, or alternatively, whether inhibition is provided by some extrinsic source. *We hypothesize that the disinhibition of pyramidal cells, suggested by Buzsaki* [80]*, mainly occurs on the axon initial segment, and the inhibition of axo-axonic cells is a necessary condition of SWRs. However, while inhibition is withdrawn from the axon initial segment, ripple frequency GABA_A_ receptor-mediated inhibition on the soma, the basal and stratum radiatum dendrites increases due to the increased firing of PV plus basket and bistratified cells. Thus, there is a temporal redistribution of inhibition over distinct subcellular domains of pyramidal cells.* This concept accounts for the remarkable synaptic target selectivity and temporal firing specificity of interneurons that we discovered. Furthermore, the results suggest that the inhibition of axo-axonic cells contributes to the consolidation of temporal sequences of pyramidal cell assemblies during SWRs, including place cell assemblies representing navigational routes.

## 4. Temporal dynamics of the same GABAergic cell types in CA1 and CA3

Most cellular elements of the circuits described above are present and similarly organized in the entire cerebral cortex, but different cortical areas differ in their temporal dynamics. For example, PV-expressing basket, axo-axonic, dendrite-innervating somatostatin-expressing and CCK-expressing basket cells innervate pyramidal cells in CA1, CA3, dentate granule cells, stellate and pyramidal cells in the entorhinal cortex. It is possible that not all principal cells in a given area that contains several types of glutamatergic cell receive equal innervation from all interneurons. In the entorhinal cortex, certain CCK-expressing basket cells innervate stellate cells projecting to other cortical areas, but not calbindin-expressing pyramidal cells [103]. The temporal dynamics of few interneuron types have been tested in more than one hippocampal area *in vivo*.

We have compared the theta- and ripple-related firing of PV-expressing basket cells in CA1 and CA3, which are organized very differently. Pyramidal cells of CA1 have limited axonal output within CA1, and their terminals frequently innervate interneurons [104], whereas pyramidal cells in CA3 have extensive interconnections via glutamatergic synapses, in addition to innervating interneurons. The latter associational connections in CA3 support the generation of gamma and high-frequency ripple oscillatory pyramidal cell firing, which are transmitted to downstream areas such as CA1 [95]. Identified PV-expressing basket cells are phase modulated in both the CA1 and CA2/3 areas by theta oscillations (figure 2). In both CA1 and CA3, the depth of theta modulation of PV-expressing basket cells is among the lowest. This might be due to phase precession during movement, i.e. backward phase shift of their preferred firing phase relative to the LFP. However, as the temporal dispersion of spikes is also apparent on a cycle-by-cycle inspection, and the depth of modulation is also low during theta in anaesthetized animals showing no phase precession, the low theta modulation is likely to be a property of this cell type. During theta, PV-expressing basket cells fire significantly later in CA2 and CA3 than in CA1 (figure 2), as measured against the CA1 pyramidal layer theta [33]. The difference in firing time during theta is paralleled by the much larger dendritic branching of PV-expressing basket cells in CA2/3 in the entorhinal input zone, the stratum lacunosum moleculare, when compared with CA1. During SWRs, PV-expressing basket cells in both CA1 and CA3 strongly increase their firing in both anaesthetized and non-anaesthetized rats (figure 2), as well as in CA1 of head-fixed awake mice [35].

Based on the selective distribution of their axons, CCK-expressing interneuron types are recognized as basket cells, proximal dendritic innervating cells (Schaffer collateral-associated cells in CA1) and perforant path-associated cells, which can be further divided on the basis of molecular expression profiles. *In vivo* recordings show several differences between the same cell type in CA1 and CA3. In general, CCK/CB1R-expressing interneurons in CA3 [32] show more cell-type-dependent diversity in their theta phase coupling than in CA1, where most of them have a mean phase preference to the ascending phase of the pyramidal layer theta LFP [38]. In CA3, the basket and the proximal dendritic layer innervating interneurons fire maximally when the local pyramidal cell firing is minimal, on the CA1 theta LFP peak. By contrast, the strongly phase-modulated perforant path-associated CCK/CB1R-expressing CA3 interneurons fire maximally 180° phase shifted, on CA1 theta troughs. Thus, CA3 pyramidal cells, unlike their CA1 counterparts receive counter-phased GABAergic input from different types of CCK/CB1R-expressing GABAergic cells. During SWRs, single CCK/CB1R-expressing interneurons in CA1 and CA3 are sometimes activated, or do not change their firing rate relative to non-SWR periods, or are inhibited [32,38]. However, in CA3, some types of CCK/CB1R-expressing interneuron were mostly activated, whereas others were mostly inhibited, showing greater diversity of participation in SWRs than in CA1.

In summary, based mostly on experiments under anaesthesia, only some of the brain-state-dependent firing dynamics of the same interneuron type can be predicted in the CA3 area from the previously described activity patterns of the same cell type in the CA1 area.

## 5. Plasticity of interneuron influence and navigation

Short- and long-term changes in the strength of both the input and the output synapses of interneurons can be influenced by pharmacological tools and synaptic activity *in vivo* [62,105] and *in vitro* [22,106–109]. These changes are probably key contributors to the formation and maintenance of cell assemblies related to navigation. Stimulation of glutamatergic pathways at theta frequency is a particularly efficacious method for changing synaptic weights [110]. Recording unidentified interneurons in novel environment showed a temporary suppression of firing [111,112], while the rats learned the new environment. A recent elegant study [113] demonstrated differential effects on the activity of individual interneurons during a goal-directed spatial navigation task involving learning. In this task, the firing patterns of pyramidal cell assemblies flickered between the representation of the new and the old maps across theta cycles; some interneurons associated their firing with the new assemblies, whereas others dissociated their activity from these. The firing associations of interneurons resulted from a local change in the efficacy of spike transmission from pyramidal cells to interneurons, either increasing or decreasing, possibly as a result of synaptic plasticity. Such association of some individual interneurons with specific cell assemblies emerged during the learning process, and remained stable in sleep and subsequent awake memory retention test [113]. It remains to be established whether the role of interneurons that increased their association with the new assembly was to suppress other assemblies, i.e. prevent their expression and/or to keep a temporal structure within the new assemblies, i.e. to maintain them, and/or prevent interference between competing assemblies by allowing their fine temporal coordination. The interneurons were recorded in and near the pyramidal cell layer with tetrodes [113] and could include about 10 distinct cell types. It will be fascinating to identify which GABA-releasing interneurons play preferential roles in the formation of new cell assemblies and learning.

## 6. Outlook

There is now ample evidence that GABAergic interneurons are key elements in the mechanisms maintaining and segregating cell assemblies as well as establishing the temporal order of assemblies during behaviours such as the sequential representation of a route. This general concept is still challenging to translate into specific cell types, circuits and synaptic links because of the limited knowledge of their spatio-temporal structure *in vivo*. Much progress is expected from experiments that can lead to the identification of interneurons during well-defined behaviours. It has already been shown in head-fixed non-anaesthetized mice that PV-expressing basket cells innervating the perisomatic region and O-LM cells innervating distal dendrites follow the same temporal sequence of firing, on average basket cells firing earlier in the cycle, during theta, gamma and ripple oscillations covering a range of 5–200 Hz [35]. Indeed, a theory posits feed-forward inhibition mediated by interneurons as a key component of maintaining temporal sequences [114]. So far, only the activity of three types of hippocampal interneuron, PV-expressing basket cells, O-LM cells and ivy cells, have been fully reported in non-anaesthetized rodents [34,35]. As more data become available, on the basis of the activity patterns of identified interneurons, it should be possible to predict the identity of the thousands of tetrode recorded interneurons in rich behavioural situations. Genetic labelling of cell types is likely to make a major contribution to deciphering the temporal dynamics of each cell type in relation to behaviour, when more selective molecular markers will become available for distinct cell types.

Some key challenges that are ripe for exploration are

— How is the segregation of the preferred firing phase of different interneuron types set up by extra- and intrahippocampal inputs during theta? The inputs from the medial septum [78] and the medial raphe nucleus [115] require further exploration in drug-free animals.
— What are the mechanisms of inhibition of some interneuron types and the presumed disinhibition of certain domains of pyramidal cells during SWRs? The case of the axo-axonic cell in CA3 is of particular interest, as SWRs are initiated by CA3 pyramidal cell population bursts, which may require the inhibition of axo-axonic cells.
— Do the rules that apply to the temporally differentiated GABA release to different domains of pyramidal cells discovered in the hippocampus also apply to other cortical areas such as the subiculum and the entorhinal cortex that contain similar cell types?
